# Genome-Wide Characterization of Long Non-Coding RNAs Identifies Candidate Regulatory Networks During Modern Maize Breeding

**DOI:** 10.3390/plants15121772

**Published:** 2026-06-08

**Authors:** Zhongyu Wang, Yang Yang, Yating He, Ning Li, Changyu Li

**Affiliations:** Biological Breeding Laboratory, Academy of Agricultural Sciences of Xinjiang Uyghur Autonomous Region, Urumqi 830091, China; wzy_caas@163.com (Z.W.); yangyang8361@163.com (Y.Y.); xjndhyt@163.com (Y.H.)

**Keywords:** long non-coding RNA, gene expression, regulatory network, maize

## Abstract

Long non-coding RNAs (lncRNAs) have emerged as important regulatory molecules in plants, but their potential roles during modern maize breeding remain largely unexplored. This study systematically characterized lncRNA expression dynamics using transcriptome data from 137 maize inbred lines from different breeding eras in China. We identified 18,023 lncRNAs transcripts, grouped by expression trends across historical breeding eras. Comparative analysis revealed 2228 differentially expressed lncRNAs transcripts (DElncRNAs) between modern (CN2000&10s) and early (CN1960&70s) accessions. By integrating WGCNA and cis-target analysis, we identified candidate lncRNAs and putative lncRNA-PCG associations that may be associated with maize plant architecture-related processes. Further, 771 DElncRNAs were predicted to be associated with 810 protein-coding genes, and these associated genes were significantly enriched in plant hormone signal transduction. Dual-luciferase reporter assays provided preliminary experimental support that lncrna.33063 and lncrna.33068 can repress the promoter activity of *ZmPIF5.2* in a heterologous transient expression system. Furthermore, we constructed a putative ceRNA-related candidate interaction network consisting of lncRNA–miRNA–mRNA triplets that include 317 candidate miRNA-lncRNA pairs and 8325 candidate miRNA-mRNA pairs, with the associated mRNAs enriched in biological processes such as morphogenesis, stimulus response, and hormone metabolism. These findings provide a set of candidate lncRNAs and lncRNA-PCG associations for future functional validation and may offer useful clues for understanding the possible roles of lncRNAs in agronomic trait-related biological processes and maize molecular breeding. Overall, this study provides candidate genetic resources and a framework for future investigation of lncRNA-associated relationships potentially related to agronomic trait variation in maize.

## 1. Introduction

Maize (*Zea mays* L.) is one of the most important cereal crops worldwide, contributing [1]. Over the past century, modern maize breeding has achieved remarkable progress and can be broadly divided into distinct breeding eras. Each breeding era has been characterized by artificial selection for key agronomic traits, including increased grain yield, improved tolerance to high planting density, and enhanced resilience to environmental stresses [2,3]. These long-term breeding efforts have profoundly shaped the maize genome, leading to the reorganization of genetic variation and the remodeling of transcriptional regulatory networks [4,5].

Long non-coding RNAs (lncRNAs) are increasingly recognized as important components of plant gene regulatory networks. Although they lack apparent protein-coding potential, lncRNAs can participate in the regulation of gene expression at multiple levels, including transcriptional and post-transcriptional regulation, and may also interact with microRNAs as endogenous target mimics or competing endogenous RNAs. In plants, lncRNAs have been associated with diverse biological processes, including growth, development, reproduction, and responses to biotic and abiotic stresses. Given that many breeding-targeted agronomic traits are complex and rely on coordinated transcriptional regulation, lncRNAs may contribute to adaptive regulatory changes associated with modern maize breeding. Therefore, characterizing lncRNA expression dynamics across breeding eras may help reveal an additional regulatory layer underlying breeding-associated adaptation. Despite considerable research on the evolutionary dynamics and selection signatures of protein-coding genes (PCGs) during maize domestication and improvement [6,7], the transcriptomic dynamics of non-coding regulatory elements, particularly lncRNAs, across modern breeding eras remain largely unexplored. A systematic investigation of lncRNAs and their potential regulatory relationships may provide new insights into the molecular mechanisms underlying maize improvement and the adaptation of modern maize germplasm to breeding targets.

Long non-coding RNAs (lncRNAs) are RNA molecules exceeding 200 nucleotides in length that do not encode proteins. They have been demonstrated to play critical regulatory roles across diverse biological processes [8,9]. In plants, accumulating evidence indicates that lncRNAs are involved in various developmental processes, respond to environmental stress, and regulate key agronomic traits [10,11,12]. For instance, certain lncRNAs have been identified as effective regulators of flowering time, root development, and abiotic stress tolerance in crops [9,11,13]. Unlike protein-coding genes, lncRNAs exhibit lower sequence conservation and display highly specific expression patterns, both temporally and spatially across various tissues [14,15]. This specificity in expression implies that lncRNAs are integral to biological evolutionary adaptation and phenotypic diversity. Therefore, an comprehensive investigation into the evolutionary dynamics of lncRNAs is essential for elucidating the regulatory mechanisms underlying crop improvement.

LncRNAs execute their biological functions through complex, hierarchical regulatory mechanisms that include both transcriptional and post-transcriptional regulation. At the transcriptional level, non-coding RNAs (ncRNAs) perform cis-regulatory roles by directly interacting with adjacent chromatin, thus modulating the expression of neighboring genes [16,17]. These target genes are often linked to quantitative trait loci (QTLs) associated with complex traits [10]. In the context of post-transcriptional regulation, the competing endogenous RNA (ceRNA) hypothesis suggests that lncRNAs can function as molecular sponges to sequester shared microRNAs (miRNAs), thus preventing the repression of their target messenger RNAs (mRNAs) [18]. This dynamic interaction among lncRNAs, miRNAs, and mRNAs forms a sophisticated trans-regulatory network that precisely regulates gene expression. Although ceRNA mechanisms have been observed during specific developmental stages or in response to stress in plants [19,20,21], a comprehensive genome-wide characterization of cis-regulatory and ceRNA networks influenced by artificial selection across various modern maize breeding eras remains to be elucidated.

While previous studies have reported the genome-wide identification of maize lncRNAs, the expression dynamics and potential regulatory associations of lncRNAs throughout the eras of modern maize breeding remain insufficiently characterized. To address this gap, we analyzed a large-scale RNA-sequencing dataset from 137 maize accessions representing different breeding eras. Using a stringent computational pipeline, we identified candidate lncRNA and characterized the expression patterns of lncRNAs and protein-coding genes across the breeding panel. We further integrated cis-association prediction, weighted gene co-expression network analysis (WGCNA), and putative ceRNA-related candidate interaction analysis to identify candidate lncRNA-PCG associations and hypothetical lncRNA–miRNA–mRNA relationships potentially related to agronomic trait-associated biological processes. These results provide a breeding-context-specific resource of candidate lncRNAs and testable hypotheses for future functional validation and maize molecular breeding-related studies.

## 2. Results

### 2.1. Systematic Identification of lncRNAs During Modern Maize Breeding

To characterize the expression features of lncRNAs across modern maize breeding eras, we performed a genome-wide systematic identification of candidate lncRNAs using published transcriptomic datasets from 137 maize inbred lines [5]. First, all sample data were aligned to the maize B73 reference genome (RefGen_V4) using HISAT2, followed by a rigorous lncRNA screening pipeline for comprehensive analysis (Figure S1A). Based on transcript length filtering, exclusion of known mRNAs and other non-coding RNAs, and integration of coding potential predictions from four tools (CPC2, CNCI, PLEK, and PFAM), a total of 18,023 candidate non-coding transcripts were initially identified (Figure S1B). To further evaluate the relationship between the lncRNAs candidates identified in this study and previously annotated maize lncRNAs, we compared our lncRNA dataset with the maize lncRNA annotations deposited in PLncDB using the same maize reference genome version [22], reducing the potential effect of genome version incompatibility. In total, our dataset and PLncDB reference dataset contained 18,023 and 32,397 lncRNAs, respectively. When exact genomic coordinate matching was applied, only 27 lncRNAs, accounting for 0.15% of our dataset, showed identical chromosome, start, and end positions between our dataset and PLncDB. Because exact coordinate matching is highly sensitive to differences in transcript boundaries, exon structures, sequencing depth, transcript assembly strategies, and annotation criteria, we further performed interval-based comparisons using BEDTools under multiple overlap thresholds. Under progressively relaxed criteria, 5869 lncRNAs, representing 32.56% of our dataset, showed at least 1 bp overlap with PLncDB lncRNAs; 4604 lncRNAs, 25.55%, showed ≥25% overlap of their genomic intervals; 4078 lncRNAs, 22.63%, showed ≥50% overlap; and 3164 lncRNAs, 17.56%, showed ≥50% reciprocal overlap (Supplementary Table S1). Using a strand-specific criterion, in which lncRNAs were considered overlapping only when at least 50% of their genomic interval overlapped with a PLncDB lncRNA on the same strand, 3230 lncRNAs, 17.92% of our dataset, overlapped with PLncDB annotations. These results indicate that exact coordinate concordance between independently assembled lncRNA datasets is limited, whereas broader interval-based comparisons reveal a larger shared fraction of previously annotated lncRNA loci. Nevertheless, a substantial proportion of lncRNA candidates identified in this study did not overlap PLncDB even under relaxed criteria, suggesting that these transcripts may represent candidate novel lncRNA loci for future validation.

**Figure 1:**
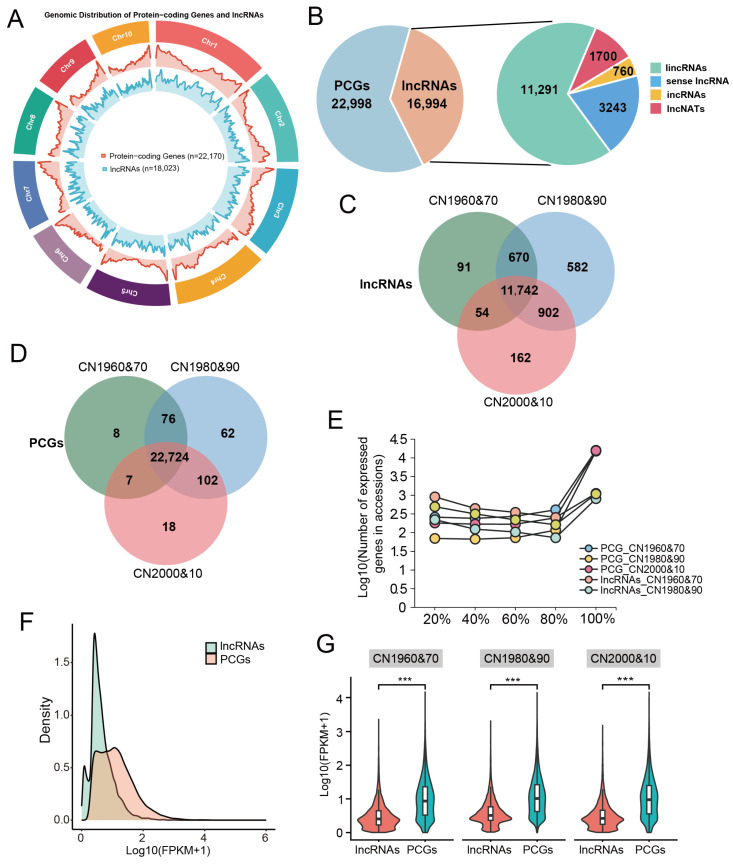
Identification and expression characteristics of lncRNAs and PCGs during maize breeding. (**A**) Genomic distribution of PCGs and lncRNAs across the 10 maize chromosomes. The outer circle represents the chromosomes, and the inner circles display the distribution densities of PCGs (red) and lncRNAs (blue). (**B**) Classification and quantitative distribution of the identified transcripts. (**C**,**D**) Venn diagrams of specific and shared transcripts across different breeding eras for (**C**) lncRNAs and (**D**) PCGs. (**E**) Conservation of lncRNAs and PCGs across maize varieties. (**F**) Overall expression level distribution of lncRNAs and PCGs. (**G**) Comparison of expression abundance for lncRNAs and PCGs across different breeding eras. *** indicates a highly significant difference between lncRNAs and PCGs (*p*-value < 0.001).

Further retention of transcripts with FPKM ≥ 0.1 in at least one sample, 16,994 expressed lncRNA candidate transcripts were obtained and widely distributed across the 10 maize chromosomes (Figure 1A). Subsequent lncRNA-related analyses were conducted at the transcript level. Based on genomic positional features, these lncRNAs were classified into four categories: 11,291 (66.4%) intergenic lncRNAs (lincRNAs), 760 (4.5%) intronic lncRNAs, 3243 (19.1%) putative sense lncRNAs, and 1700 (10.0%) putative antisense lncRNAs (Figure 1B). For background comparison, we performed parallel analysis of protein-coding genes (PCGs) and identified 22,998 expressed PCGs (Figure 1A). To evaluate the reliability of the identified lncRNAs candidates, we further analyzed their structural and expression features. In comparison to PCGs, the identified lncRNAs generally exhibited shorter transcript lengths, fewer exons, and shorter open reading frames, which are consistent with typical characteristics of plant lncRNAs (Figure S2).

To evaluate lncRNA expression dynamics across modern maize breeding eras, we categorized the inbred lines into three distinct breeding eras (CN1960&70, CN1980&90, and CN2000&2010) for comparative analysis. This classification was based primarily on the breeding or release periods of the inbred lines in China. We detected 12,557, 13,896, and 12,860 expressed lncRNAs in these respective periods (Figure 1C), which were utilized as candidate sets for further analyses. In contrast, the corresponding eras exhibited 22,815, 22,982, and 22,964 expressed PCGs (Figure 1D). Notably, 98.8% (22,742) of PCGs were expressed across all three breeding eras, whereas only ~69.0% (11,742) of lncRNAs were shared among the three eras, indicating that lncRNAs showed greater expression variability PCGs in this breeding-era panel.

Further analysis of expression frequency at the individual inbred line revealed that only 4.7–6.6% of lncRNAs were detected in all inbred lines within each group (1124/16,994 for CN1960&70; 809/16,994 for CN1980&90; 1083/16,994 for CN2000&2010). In contrast, PCGs showed much higher expression rates of 65.5–69.4% (15,965/22,998, 15,071/22,998, and 15,684/22,998 for corresponding eras, respectively) (Figure 1E). Most lncRNAs were expressed in less than 20% of maize inbred lines within each group (14,821/16,994, 15,884/16,994, and 15,211/16,994 for the corresponding eras), indicating more restricted or genotype-dependent expression patterns relative to PCGs (Figure 1E). Additionally, overall expression levels of lncRNAs were significantly lower than those of PCGs (Figure 1F,G).

To further investigate whether the identified lncRNAs were co-localized with genomic regions showing previously reported selection signals in maize breeding-related comparisons, we compared the genomic coordinates of lncRNA loci with previously published maize selection signal regions [3]. A total of 17,173 unique lncRNA loci were included in this analysis. Among them, 934 lncRNAs showed at least 1-bp overlap with the selection regions identified in the CN1980&90 vs. CN1960&70 comparison, and 903 lncRNAs were completely contained within these regions. For the CN2000&10 vs. CN1960&70 comparison, 1076 lncRNAs overlapped with selection regions, including 1017 that were completely contained within selection regions (Table 1). For the CN2000&10 vs. CN1980&90 comparison, 846 overlapping lncRNAs were detected, of which 799 were completely contained within selection regions. In addition, 1935 lncRNAs overlapped with the Ex-PVP vs. Public-US selection regions, and 1906 of them were completely contained within these regions. To assess whether these overlaps exceeded random genomic expectations, we conducted a permutation-based enrichment analysis. The positions of lncRNA loci were randomly shuffled 10,000 times while preserving chromosome assignment and locus length distribution, and overlaps with each set of selection regions were recalculated to generate a null distribution. Significant enrichment of lncRNA loci in selection regions was observed for three comparisons: CN1980&90s vs. CN1960&70, CN2000&10 vs. CN1960&70, and CN2000&10 vs. CN1980&90, with empirical *p*-values of 0.009987, 0.00987001, and 0.00029997, respectively. In contrast, no significant enrichment was detected for the Ex-PVP vs. Public-US comparison (Table 1). These findings indicate that lncRNA loci are not uniformly enriched across all selection datasets but show statistically supported co-localization with selection signal regions in specific breeding-era comparisons. Thus, these overlaps should be interpreted as supportive evidence for possible associations between candidate lncRNA loci and breeding-related genomic regions, rather than as direct evidence for selection on individual lncRNAs or their functional involvement in maize improvement.

### 2.2. Global Expression Patterns and Differential Expression Analysis of lncRNAs and PCGs

**Figure 2:**
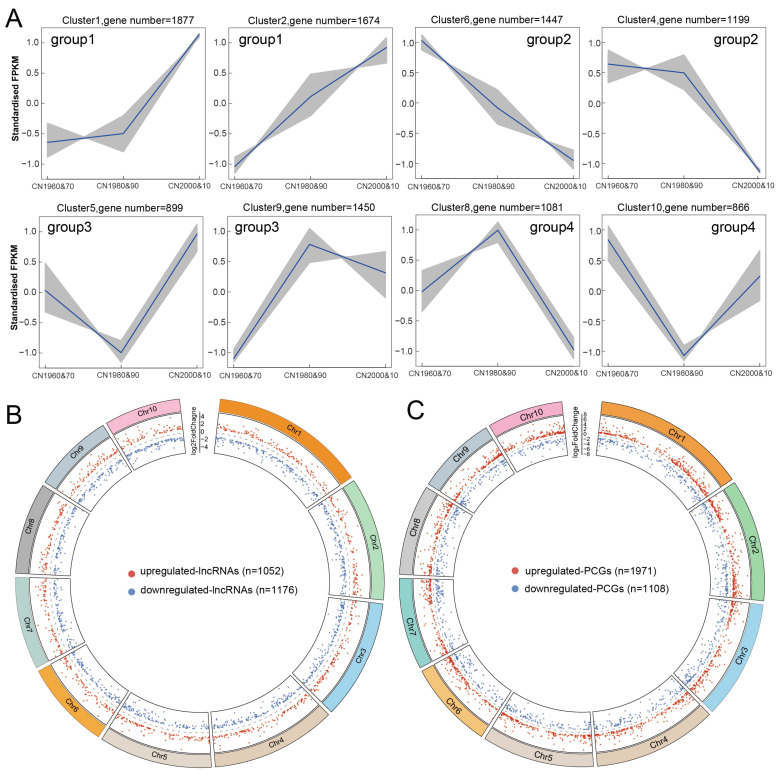
Evolutionary dynamics and differential expression analysis of lncRNAs and PCGs during modern maize breeding. (**A**) Spatiotemporal expression patterns of lncRNAs across the three breeding eras. (**B**,**C**) Circos plots illustrating the genomic distributions of differentially expressed lncRNAs (**B**) and PCGs (**C**) between the early and modern breeding eras. The outer circle represents the 10 maize chromosomes, and the scatter plots show the log_2_ (fold change) in expression levels for each transcript in the CN2000&10 vs. CN1960&70 comparison.

To investigate the expression dynamics of lncRNAs across modern maize breeding eras, we performed K-means clustering analysis on expressed lncRNAs across the three breeding-era groups. The results revealed that all expressed lncRNAs were categorized 10 expression clusters, which were further grouped into four major expression patterns: progressively increased expression from early to recent breeding-era groups (Group 1), progressively decreased expression (Group 2), genes with fluctuating expression patterns but exhibiting higher expression levels in the recent breeding stages (CN2000&2010) than in the early stages (CN1960&70) (Group 3), and genes with fluctuating expression patterns but lower expression levels in the recent stages than in the early stages (Group 4) (Figure 2A).

To further compare lncRNA and PCG expression between early- and recent-era maize inbred lines, we conducted differential expression analysis between the early (CN1960&70) and recent (CN2000&2010) inbred lines. Genes with |log_2_ (Fold Change)| ≥ 1 were defined as differentially expressed lncRNAs (DElncRNAs) and differentially expressed PCGs (DEPCGs). We finally identified 2228 DElncRNAs, including 1052 upregulated and 1176 downregulated (Figure 2B, Supplementary Table S2), as well as 3079 DEPCGs were identified, of which 1971 were upregulated and 1108 were downregulated (Figure 2C, Supplementary Table S3).

### 2.3. Putative Cis-Associations Between Differentially Expressed lncRNAs and Neighboring Protein-Coding Genes Across Maize Breeding Eras

**Figure 3:**
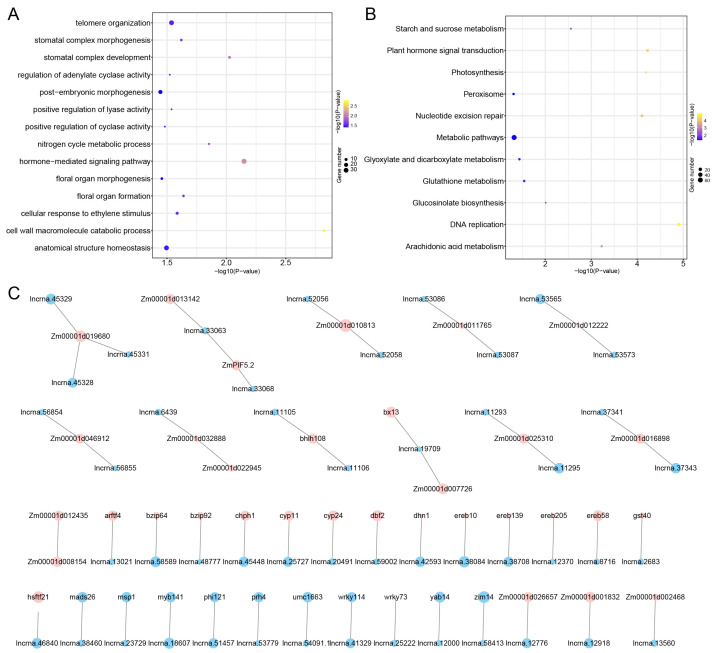
Functional enrichment analysis and cis-regulatory networks of DElncRNAs and their candidate cis-associated target genes. (**A**) Gene Ontology (GO) enrichment analysis of the cis-target PCGs of DElncRNAs. (**B**) KEGG pathway enrichment analysis of the cis-target PCGs. (**C**) Core candidate cis-regulatory network modules between DElncRNAs and their target PCGs. Blue nodes represent DElncRNAs, and pink nodes denote their candidate cis-associated target genes.

To explore putative associations between DElncRNAs and neighboring protein-coding genes, we predicted candidate cis-associated genes by identifying colocalized genes within 100 kb upstream or downstream of each DElncRNA. In total, 2101 DElncRNAs were located near 9676 neighboring protein-coding genes. By integrating these results with the differential expression analysis of protein-coding genes (DEPCGs), we ultimately identified 771 DElncRNAs that were putatively cis-associated with 810 DEPCGs (Supplementary Table S4). GO and KEGG enrichment analyses were performed on these candidate cis-associated DEPCGs. The GO enrichment results showed that the target genes were mainly enriched in biological processes related to cell wall macromolecule catabolism, hormone-mediated signaling pathways, nitrogen cycle metabolic processes, floral organ formation, and post-embryonic morphogenesis (Figure 3A). KEGG analysis further confirmed that these target genes were significantly involved in several key pathways, including plant hormone signal transduction, photosynthesis, starch and sucrose metabolism, and glutathione metabolism (Figure 3B). Notably, 36 DEPCGs were enriched in the plant hormone signaling pathways, including auxin, brassinosteroid, and ethylene signaling pathways, such as *ARF4*, *Bx3*, *EREB205*, and *ZmPIF5.2*. Based on these candidate relationships, we constructed a core predicted lncRNA-mRNA cis-association network for the plant hormone signal transduction pathway (Figure 3C).

To refine candidate cis-associated lncRNA-mRNA pairs, we applied an expression-correlation filter to the candidate lncRNA-mRNA pairs identified within the 100 k bp genomic window. Pearson correlation analysis was performed across all RNA-seq samples, and only pairs with |Pearson’s *r*| ≥ 0.6 and *p*-value < 0.05 were retained. Following this filtering process, 424 candidate lncRNA-mRNA pairs supported by both genomic proximity and expression correlation (Supplementary Table S5). Among these high-confidence regulatory pairs, *ZmPIF5.2* (*Zm00001d013130*) was selected for further investigation due to its previous established roles in both conserved and unique molecular mechanisms governing light signal transduction and photomorphogenesis in maize [23]. In the predicted lncRNA-mRNA association network, *ZmPIF5.2* was associated with two adjacent lncRNAs, lncrna.33063 and lncrna.33068, based on both genomic proximity and expression correlation.

**Figure 4:**
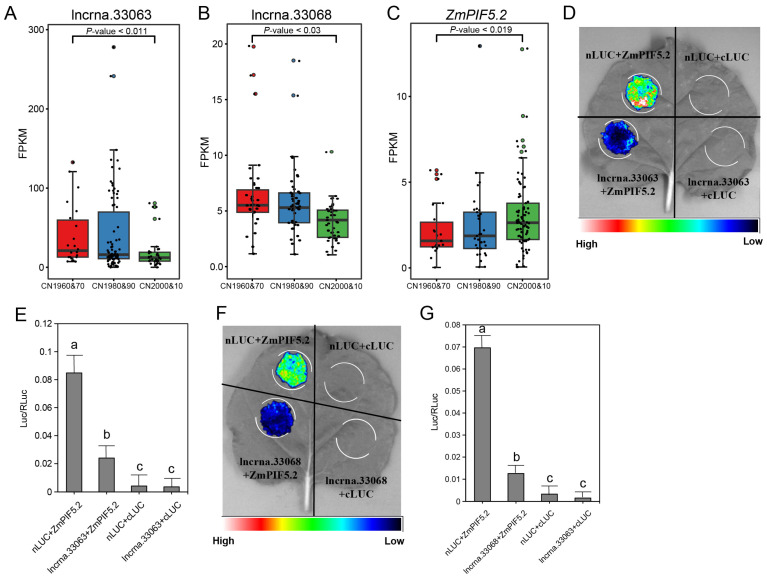
Expression profiles and regulatory validation of lncrna.33063, lncrna.33068, and their target gene *ZmPIF5.2*. (**A**–**C**) Box plots showing expression levels of (**A**) lncrna.33063, (**B**) lncrna.33068, and (**C**) their target gene *ZmPIF5.2* across three maize breeding eras (CN1960&70, CN1980&90, and CN2000&10). (**D**) Schematic diagram of the dual-luciferase reporter assay designed to detect transcriptional activation of the *ZmPIF5.2* promoter by lncrna.33063. (**E**) Quantitative analysis of dual-luciferase reporter activities in *Nicotiana benthamiana* leaves co-expressing *pZmPIF5.2*-LUC with lncrna.33063 or corresponding control constructs. Data were presented as mean ± standard deviation from three independent biological replicates. Different letters indicate significant differences (*p*-value < 0.05, one-way ANOVA with Tukey’s HSD test). (**F**) Dual-luciferase reporter assay to assess transcriptional activation of the *ZmPIF5.2* promoter by lncrna.33068. (**G**) Quantitative analysis of relative luciferase activities (Luc/RLuc ratio) from fluorescence imaging in *N. benthamiana* leaves.

Transcriptome analysis showed that *ZmPIF5.2* was significantly upregulated during modern maize breeding, whereas lncrna.33063 and lncrna.33068 were notably downregulated, suggesting a potential negative association between these lncRNAs and *ZmPIF5.2* expression (Figure 4A–C). To test whether these lncRNAs could affect *ZmPIF5.2* promoter activity, we performed dual-luciferase reporter assays in *Nicotiana benthamiana*. The *ZmPIF5.2* promoter-driven reporter was co-expressed with lncrna.33063, lncrna.33068, or the corresponding empty-vector control. Compared with the empty-vector control, both lncRNAs significantly reduced *ZmPIF5.2* promoter activity in this heterologous transient expression system (Figure 4D–G). These results suggest that lncrna.33063 and lncrna.33068 can repress *ZmPIF5.2* promoter activity in a heterologous assay; however, they do not establish direct promoter interaction, chromatin association, or endogenous regulation in maize. Further in planta validation will be required to confirm whether these lncRNAs regulate *ZmPIF5.2* under native maize conditions. Moreover, the reduced expression of lncrna.33063 and lncrna.33068 in modern breeding lines may also represent an indirect consequence of breeding-associated selection on light responsiveness, compact plant architecture, or stress-adaptation pathways, rather than direct selection on these lncRNA loci themselves. Therefore, these results should be interpreted as evidence for a candidate lncRNA-*ZmPIF5.2* association regulatory association potentially linked to the remodeling of light- and hormone-related regulatory networks during modern maize breeding.

### 2.4. Co-Expression Network Analysis Reveals the Roles of Long Non-Coding RNAs and PCGs During Modern Maize Breeding

**Figure 5:**
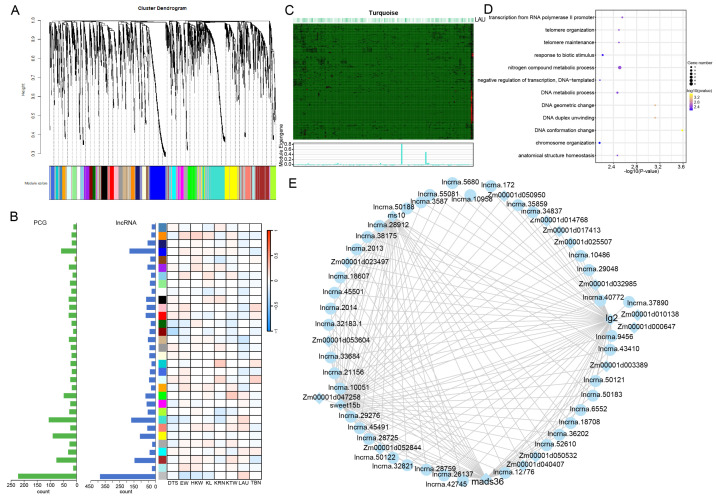
Weighted gene co-expression network analysis (WGCNA) identifies core modules and lncRNA-PCG networks associated with agronomic traits. (**A**) Hierarchical clustering dendrogram of DElncRNAs and DEPCGs. (**B**) Quantitative distribution of lncRNAs and PCGs in each module and their correlations with agronomic traits. The left bar chart shows the number of PCGs (green) and lncRNAs (blue) in each module. The right heatmap shows the correlation coefficients (Pearson’s r) between the module eigengenes and eight selected agronomic traits. Red and blue denote positive and negative correlations, respectively. (**C**) Expression profile of the Turquoise module, which is significantly correlated with leaf angle (LAU). (**D**) Gene Ontology (GO) enrichment analysis of the hub genes within the Turquoise module. (**E**) Core regulatory network of the Turquoise module.

To explore co-expression patterns potentially associated with selected agronomic traits, we performed a weighted gene co-expression network analysis (WGCNA) utilizing expression data from 2228 DElncRNAs and 3079 DEPCGs. Hierarchical clustering identified 33 co-expression modules (Figure 5A), and the distribution of lncRNAs and PCGs within each module depicted in Figure 5B. The turquoise, blue, and brown modules contained relatively higher number of lncRNAs and PCGs compared to other modules. Although lncRNAs accounted for a smaller fraction in most modules, the co-expressed PCGs provided a basis for exploring putative functional associations of lncRNAs through annotated protein-coding genes.

To assess potential module–trait associations, we correlated module eigengenes with eight agronomic traits available for the studied maize panel: days to silking (DTS), ear weight (EW), hundred-kernel weight (HKW), kernel thickness width (KTW), kernel length (KL), leaf angle upper (LAU), kernel row number (KRN), and tassel branch number (TBN). These traits represent flowering time-, plant architecture-, and yield-related agronomic characteristics and have been previously reported to be relevant to maize breeding-era comparisons [5]. Modules with significant module–trait correlations were identified using a threshold of *p*-value < 0.05. The turquoise module was positively correlated with LAU (*r* = 0.17, *p*-value = 0.005), the brown module was negatively correlated with TBN (*r* = −0.19, *p*-value = 0.003), and the dark orange module was positively correlated with KRN (*r* = 0.22, *p*-value = 0.001) (Figure 5C and Figure S4). Additional significant correlations were detected between the light cyan and tan modules and LAU, as well as between the dark red module and DTS. Although these module–trait correlations were statistically significant, their magnitudes were modest (|*r*| = 0.17–0.22), suggesting that the corresponding modules explain only a small proportion of phenotypic variation. Thus, these modules should be regarded as putative trait-associated co-expression modules, rather than as evidence for established regulatory effects on agronomic traits. We therefore used these modules, together with functional enrichment results and known trait-related genes, to prioritize candidate lncRNA-PCG co-expression associations for future validation.

To assess the preservation of the WGCNA modules, we conducted a module preservation analysis utilizing the modulePreservation function in WGCNA. A total of 33 modules were assessed using Zsummary and medianRank statistics (Supplementary Table S6). Most principal modules showed moderate to strong preservation, suggesting that the co-expression structure was generally robust across different sample subsets. Notably, modules showing significant but weak correlations with selected agronomic traits also showed evidence of preservation. For example, the turquoise module, which exhibited a positive correlation with LAU, showed strong preservation with a Zsummary value of 30.84. Similarly, the brown module, negatively correlated with TBN, and the dark orange module, positively correlated with KRN, also demonstrated strong preservation, with Zsummary values of 17.47 and 11.70, respectively. These findings support the stability of the inferred co-expression structure, but they should not be interpreted as evidence of direct trait-regulatory effects, given the modest module–trait correlations.

Among the preserved modules, the turquoise module showed a weak but significant positive correlation with LAU. Varieties with smaller leaf angles tended to show lower expression levels of lncRNAs and PCGs in this module (Figure 5C). Based on intramodular connectivity (ME > 0.8), we identified 48 candidate hub genes in this putative LAU-associated module. These genes were mainly enriched in DNA modification, metabolic processes, and stomatal complex development pathways (Figure 5D). Subsequently, a putative LAU-related co-expression network was then constructed, which included several highly connected genes such as *Liguleless2* (*lg2*), *mads36*, *ms10*, and *sweet15b* (Figure 5E). Previous studies have indicated that *lg2* mutant homozygous single-cross hybrids exhibit upright leaves, leading to a 40% yield increase compared to controls [24]. Thus, the 52 lncRNAs and PCGs co-expressed with *lg2* in this network may represent candidate components associated with leaf angle-related expression variation. However, these relationships are based on co-expression and prior functional annotation, and their relevance to leaf angle regulation will require further experimental validation.

### 2.5. Construction and Analysis of the Putative ceRNA-Associated Network

The competing endogenous RNA (ceRNA) hypothesis proposes that different RNA molecules may interact through shared miRNA response elements. In plants, lncRNAs have been reported to act as potential miRNA decoys or target mimics in certain regulatory contexts. To explore putative ceRNA-related associations, we constructed a miRNA-centered predicted ceRNA-associated network based on bioinformatic prediction of lncRNA-miRNA and miRNA-mRNA binding sites. This network included 8325 predicted miRNA-mRNA pairs and 317 predicted miRNA-lncRNA pairs. By further integrating lncRNA and mRNA expression profiles, we identified 1623 candidate DElncRNA–miRNA–DEPCG triplets in which both DElncRNAs and DEPCGs were coordinately downregulated, and 2728 candidate triplets in which both were coordinately upregulated (Supplementary Table S7).

**Figure 6:**
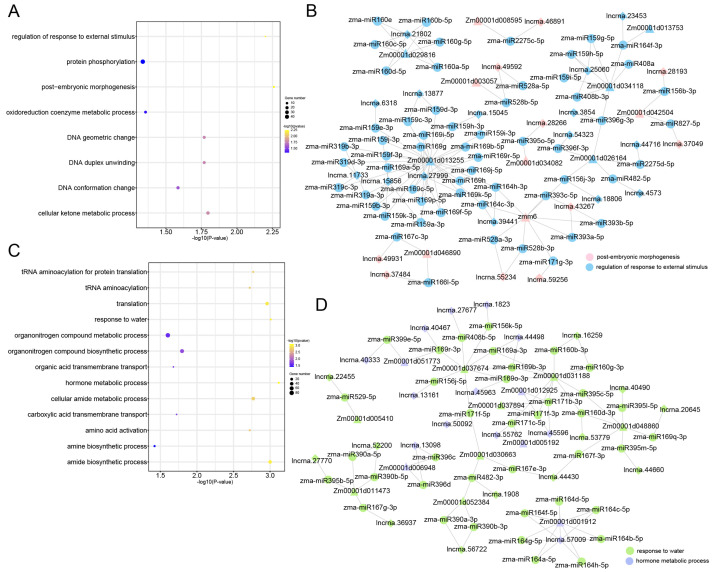
Construction and functional enrichment analysis of the putative ceRNA-associated network. (**A**) Gene Ontology (GO) enrichment analysis of target PCGs in the co-upregulated ceRNA network. (**B**) Representative co-upregulated ceRNA subnetwork. Triangles, circles, and diamonds represent lncRNAs, miRNAs, and mRNAs, respectively. (**C**) GO enrichment analysis of target PCGs in the co-downregulated ceRNA network. (**D**) Representative co-downregulated putative ceRNA-associated subnetwork. Triangles, circles, and diamonds represent lncRNAs, miRNAs, and mRNAs, respectively.

To assess the potential biological relevance of these predicted candidates, we performed enrichment analysis of the predicted target PCGs involved in these networks. In the upregulated candidate network, the predicted target PCGs were mainly enriched in pathways such as post-embryonic morphogenesis, regulation of response to external stimuli, cellular ketone metabolic process, and protein phosphorylation (Figure 6A). We therefore further examined the candidate triplets associated with the post-embryonic morphogenesis pathway. This predicted interaction subset consisted of five lncRNAs, nine miRNAs, and one predicted target PCG (Figure 6B). Specifically, five candidate lncRNAs (lncrna.18806, lncrna.39441, lncrna.59256, lncrna.43267, and lncrna.55234) were predicted to share miRNA regulators with Zm00001d031620 (*zmm6*) through different subsets of nine miRNAs (zma-miR156j-3p, zma-miR164c-3p, zma-miR164h-3p, zma-miR171g-3p, zma-miR393a-5p, zma-miR393b-5p, zma-miR393c-5p, zma-miR528a-3p, and zma-miR528b-3p). *Zmm6* belongs to the *AGL2* MADS-box subfamily, and its expression is primarily restricted to developing inflorescences and kernels [25].

In the downregulated candidate ceRNA-associated network, the predicted target PCGs were predominantly enriched in pathways related to hormone metabolic processes, responses to water, amide biosynthetic processes, and cellular amide metabolic processes (Figure 6C). Based on these enrichment findings, our analysis further concentrated on predicted target genes associated with the response to water and hormone metabolic process pathways. For the response to water pathway, lncrna.45596 was predicted to share miRNA regulators, including zma-miR171f-3p and zma-miR171b-3p, with the predicted target gene Zm00001d037894 (*ZmDHN1*) (Figure 6D). *ZmDHN1* is a known drought-responsive gene in maize [26]. For the hormone metabolic process pathway, a candidate interaction subset consisting of seven lncRNAs, eight miRNAs, and one predicted target mRNA was identified (Figure 6D). In this subset, the predicted target gene Zm00001d037674 (*TAR3*) encodes a key component of the IAA biosynthetic pathway.

Overall, these analyses identified computationally predicted candidate lncRNA–miRNA–mRNA triplets potentially related to plant development, stress response, and agronomic trait variation across maize breeding-era groups. However, these putative ceRNA-like relationships are inferred from predicted shared miRNA targeting and coordinated lncRNA-mRNA expression patterns. These data do not establish bona fide ceRNA regulation, particularly because matched miRNA expression profiles were not available. Further validation using matched small RNA-seq, degradome analysis, and molecular experiments will be required to confirm miRNA expression, cleavage or binding relationships, physical interactions, and biological functions.

## 3. Discussion

We characterized the expression dynamics and putative regulatory associations of maize lncRNAs across breeding-era groups, suggesting that some lncRNAs may be associated with transcriptional and agronomic differences observed among modern maize breeding materials. Because maize lncRNA annotations remain incomplete, we applied a stringent multi-step pipeline integrating transcript annotation comparison, length filtering, CNCI, CPC2, PLEK, and Pfam/HMMER analyses to generate a conservative lncRNA candidate set for downstream analyses. The limited overlap between our lncRNA catalog and PLncDB should therefore be interpreted cautiously, as it likely reflects not only transcript boundary differences, but also the strong tissue-, genotype-, developmental stage-, and condition-specific expression of lncRNAs, together with differences in data type, transcript assembly, and filtering strategies. In particular, the use of poly(A)-selected RNA-seq together with a stringent multi-step filtering framework may have reduced overlap with existing annotations while increasing confidence in the retained candidate transcripts. Several limitations should also be acknowledged. Because the poly(A)-selected RNA-seq data mainly captured polyadenylated transcripts, non-polyadenylated plant lncRNAs were likely underrepresented. In addition, the requirement for at least two exons may have excluded genuine mono-exonic lncRNAs, and sense/antisense classification should be interpreted cautiously when strand-specific information is limited. Therefore, the relatively low overlap with PLncDB should not be taken as evidence of a high false-positive rate, but rather as an indication of the context-dependent and still incomplete nature of maize lncRNA annotation. Future studies using rRNA-depleted total RNA-seq, strand-specific RNA-seq, and long-read sequencing will be important for building a more complete maize lncRNA catalog.

Our analysis revealed marked expression variability of maize long non-coding RNAs (lncRNAs) across different breeding eras (CN1960&70, CN1980&90, CN2000&2010). Approximately 69.0% of lncRNAs were expressed across all three breeding-era groups, while the proportion of protein-coding genes (PCGs) exhibited a continuity rate of 98.8% (Figure 1C–E). Moreover, the majority of lncRNAs (>85%) were found to be specifically expressed in a limited number of inbred lines, with their overall expression levels being significantly lower than those of PCGs (Figure 1F,G). These results indicate that lncRNAs show greater expression variability and genotype specificity than PCGs in the analyzed maize panel. Such expression patterns may reflect the generally low expression level, context-dependent regulation, and incomplete annotation of plant lncRNAs, and may also be associated with genotype- or breeding-era-related transcriptomic differences [27]. However, the current analysis does not establish whether these expression differences were directly shaped by environmental adaptation or artificial selection. This observation aligns closely with previously documented expression dynamics of PCGs [5]. The observed variety-specific expression of lncRNAs therefore should be interpreted as evidence for candidate lncRNA-associated transcriptomic variation, rather than as direct evidence for functional contributions to maize genetic diversity or agronomic trait regulation. Future studies integrating population-genetic analyses, eQTL mapping, genome-wide association studies, and trait QTL information will be needed to further evaluate the relationships between lncRNA expression variation and agronomic trait-associated loci.

Through cis-target prediction, we identified 771 differentially expressed lncRNAs (DElncRNAs) that were predicted to be located near 810 differentially expressed protein-coding genes (DEPCGs). GO and KEGG enrichment analyses showed that these putative cis-associated DEPCGs were significantly enriched in phytohormone signal transduction, photosynthesis, and starch metabolism (Figure 3A,B). Notably, the phytohormone signal transduction pathway was enriched with key factors such as ARF4, Bx3, EREB205, and ZmPIF5.2, which are involved in classical regulatory pathways such as those for auxin, brassinosteroid, and ethylene (Figure 3C). Since phytohormones play central roles in plant architecture establishment, organogenesis, stress responses, and yield formation, these DElncRNAs may represent candidate lncRNA associated with hormone-related transcriptional variation across maize breeding-era groups [28]. Previous studies have suggested that some plant lncRNAs may influence or be associated with the expression of neighboring genes through cis-related mechanisms [29]. However, the cis relationships identified here are based primarily on genomic proximity and coordinated differential expression, and therefore should be interpreted as putative lncRNA-mRNA cis-associations rather than confirmed regulatory interactions. Thus, the predicted lncRNA-mRNA cis-association network provides candidate resources for future functional validation of non-coding RNA-associated transcriptional variation in maize, rather than direct evidence for their contribution to maize trait improvement.

It should also be noted that the decreased expression of lncrna.33063 and lncrna.33068 in modern breeding lines does not by itself indicate direct selection on these lncRNA loci. PIF family proteins are classically known to accumulate in darkness and promote skotomorphogenesis and etiolation, but they have also been reported to participate broadly in light signaling, phytohormone responses, shade avoidance, plant architecture, and stress-related pathways. The opposite expression patterns of *ZmPIF5.2* and its two associated lncRNAs may therefore reflect broader breeding-era-associated remodeling of light- and hormone-related transcriptional networks. One possible interpretation is that the reduced expression of lncrna.33063 and lncrna.33068 arose indirectly in association with breeding for altered light responsiveness, compact plant architecture, high-density planting adaptation, or stress-related traits. In addition, the co-expression evidence in this study was derived from whole-seedling transcriptomes of maize. Although seedlings encompass developing young leaves, leaf sheaths, and the shoot apical meristem (SAM), bulk RNA-seq data lack the cellular resolution to disentangle expression signals from specific micro-tissues. Consequently, observed co-expression may reflect tissue composition rather than true regulatory coordination within a single cell type. High-resolution spatial transcriptomics and RNA FISH are therefore warranted to validate cell-type-specific co-localization and refine these co-expression relationships. Although the dual-luciferase reporter assays provided experimental support that these two lncRNAs can repress *ZmPIF5.2* promoter activity in a heterologous transient system, these data do not establish in planta regulatory effects, direct targets of artificial selection, or causal contributions to compact growth habit. Further in planta functional validation, population-genetic analysis, and phenotypic characterization of lncRNA perturbation lines will be necessary to clarify their biological roles and their possible relevance to breeding-era-associated phenotypic variation.

The possible relationship between lncRNA expression changes and breeding-associated selection is particularly relevant to high-density planting adaptation. During modern maize breeding, compact plant architecture, especially reduced leaf angle, has been an important breeding objective for improving canopy light interception and population yield under dense planting. In this study, the turquoise module showed a weak but significant correlation with upper leaf angle and contained the known leaf-angle regulator *lg2* [30,31]. Several lncRNAs were co-expressed with *lg2* and other plant architecture-related genes in this module, suggesting that they may represent candidate co-expression components within a putative leaf-angle-related module. In addition, some lncRNA loci overlapped with previously reported selection signal regions. Together, these observations suggest that a subset of breeding-era-associated lncRNA expression differences may be associated with plant architecture-related genomic regions or expression modules, including those potentially relevant to high-density planting adaptation. However, these relationships are inferred from co-expression and genomic co-localization and therefore should be interpreted as correlative rather than causal. It is also important to note that the module–trait correlations identified here were generally weak (|*r*| = 0.17–0.22), indicating that the corresponding modules explain only a limited proportion of phenotype variation. This is not unexpected, because agronomic traits such as plant architecture and yield-related traits are complex quantitative traits shaped by multiple loci, developmental processes, environmental effects, and genotype-by-environment interactions. In addition, WGCNA captures coordinated transcript variation, which does not necessarily correspond directly to phenotype variation. Thus, these weak but statistically significant correlations should be interpreted as preliminary co-expression-based evidence for possible trait associations, rather than direct genotype–phenotype relationships. Population structure, breeding group effects, and lineage-specific expression patterns may also influence both transcript abundance and agronomic traits in maize diversity panels. Because these factors were not explicitly modeled in the current module–trait analysis, it remains possible that some observed expression differences or module–trait associations partly reflect lineage structure or breeding group composition rather than breeding-associated selection itself. Future studies incorporating population structure, kinship, breeding group information, multi-environment phenotype, and genetic perturbation experiments will be required to distinguish breeding-era-associated expression variation from lineage-associated effects and to validate the biological relevance of the candidate modules.

The ceRNA hypothesis proposes that different RNA molecules may competitively bind shared miRNA response elements, thereby forming complex post-transcriptional regulatory networks [18]. In plants, accumulating evidence supports the idea that lncRNAs can function as miRNA decoys or sponges, buffering the inhibitory effect of miRNAs on target genes and thereby participating in processes such as flowering, root development, and stress response [32,33]. Based on this hypothesis, we constructed a predicted regulatory-associated interaction network containing 8325 miRNA-mRNA pairs and 317 miRNA-lncRNA pairs, and identified 4351 candidate DElncRNA-miRNA-DEPCG triplets. Among these, the upregulated putative ceRNA-associated network was enriched in processes such as post-embryonic morphogenesis (Figure 6A). Further analysis revealed that five lncRNAs were predicted to share putative miRNA-mediated relationships with nine miRNAs and the predicted target gene Zm00001d031620 (*zmm6*) (Figure 6B). *Zmm6* belongs to the *AGL2* subfamily of MADS-box genes, and its expression is mainly detected in developing inflorescences and kernels, functioning closely with reproductive development [25,34]. However, these inferred relationships should be interpreted cautiously. The ceRNA-associated network in this study was based primarily on computational prediction of shared miRNA-binding sites with positive lncRNA–mRNA expression correlations. Because matched miRNA expression profiles and degradome data were unavailable, the identified candidate lncRNA–miRNA–mRNA triplets should be considered candidate interactions rather than experimentally validated ceRNA regulatory relationships. Future studies integrating small RNA sequencing, degradome sequencing, and experimental validation approaches, such as dual-luciferase reporter assays, RLM-5′ RACE, RNA pull-down, and genetic perturbation, will be required to confirm these putative ceRNA interactions. This study systematically characterized the expression patterns and breeding-era-associated variation in maize lncRNAs using transcriptome data from different breeding-era groups, and identified candidate lncRNA–PCG associations and putative ceRNA-like relationships through cis-target prediction and lncRNA-associated interaction network analyses. These findings provide a breeding-context-specific lncRNA resource and testable hypotheses for investigating the possible roles of non-coding RNAs in maize improvement-associated transcriptional variation. Future studies using gene-editing approaches, together with multi-environment phenotyping and analyses across diverse genetic backgrounds, will be needed to test the biological relevance of selected lncRNAs and to evaluate their possible associations with yield-related and stress-related trait variation in maize.

## 4. Materials and Methods

### 4.1. RNA-Seq Data Processing and Quality Control

The 137 maize inbred lines were assigned to three breeding-era groups according to the classification of the original dataset, which was primarily based on their breeding/release periods in China rather than pedigree relationships. These groups represent historical stages of modern maize breeding: CN1960&70s, CN1980&90s, and CN2000&10s. Seedlings of the 137 inbred lines were grown in a growth greenhouse (16 h of light/8 h of dark, light intensity of 200 mmol photons m^−2^s^−1^, at ~60% humidity) with daytime temperature set at 28 °C and night temperature set at 22 °C. When the seedlings reached the V2 stage, all aboveground plant parts were collected from 3 replicates of 3 plants. Seedlings were collected in liquid nitrogen and stored at −80 °C before RNA extraction. Total RNA was extracted using the TRIzol reagent (Life Technologies, Carlsbad, CA, USA) according to the manufacturer’s protocol. RNA-seq libraries were constructed for Illumina protocols using the manufacturer-specified methods (Illumina, San Diego, CA, USA) and sequenced to generate 150-nucleotide paired-end reads on the Illumina HiSeq 2000 platform (Illumina, San Diego, CA, USA). The raw reads from the published RNA-seq data used in this study were obtained from PRJNA957932 [5]. According to the dataset metadata, the libraries were generated using a poly(A)-selection/enrichment strategy prior to Illumina sequencing. Therefore, the transcriptome assemblies and lncRNA identification in this study were primarily based on polyadenylated transcripts. Trimmomatic (v0.39) was used to eliminate adapter sequences and low-quality reads [35]. The clean reads were mapped to the maize reference genome (B73 RefGen_v4) using HISAT2 (v2.2.1) with default parameters [36]. Samtools (v1.10) was employed to convert the resulting SAM files to BAM format and sort them [37]. Read counts for lncRNAs and protein-coding genes were generated using featureCounts from the Subread package (v2.0.1) [38].

### 4.2. Genome-Wide Identification of Long Non-Coding RNAs

To identify high-confidence long non-coding RNAs (lncRNAs) from the assembled maize transcriptomes, a highly stringent multi-step computational method was applied. First, transcript assembly was then performed separately for each sample using Cufflinks (v2.2.1). The resulting transcript assemblies were subsequently merged to generate a unified, non-redundant transcript set, which was used for downstream lncRNA identification. Transcripts overlapping known protein-coding genes (PCGs) or annotated non-coding RNAs were removed using gffcompare (v0.11.2) [39]. Second, the remaining unannotated transcripts were filtered based on length and exon count, in which only transcripts with a length of ≥200 nucleotides and containing at least two exons were retained to obtain a conservative set of high-confidence lncRNAs and reduce potential transcriptional noise. Third, to stringently eliminate transcripts with protein-coding potential, four independent prediction tools were applied, including the Coding-Non-Coding Index (CNCI) [40], Coding Potential Calculator 2 (CPC2) [41], Predictor of Long Non-Coding RNAs and mRNAs based on an Improved k-mer Scheme (PLEK) [42], and a Pfam protein database scan using HMMER (v3.3) [43]. CPC2, CNCI, and PLEK were used to predict coding potential based on sequence features, whereas Pfam was used to identify transcripts containing conserved protein domains. Transcripts predicted as coding by any of these tools or containing known protein domains were removed. Only transcripts consistently predicted as non-coding by all four methods were defined as high-confidence lncRNAs and retained for subsequent analyses.

### 4.3. Permutation-Based Enrichment Analysis of lncRNA Overlap with Selection Regions

To determine whether lncRNA loci were significantly enriched in previously reported breeding-associated selection regions, we performed a permutation-based enrichment analysis. Genomic coordinates of lncRNA loci and selection regions were converted into BED format. The observed number of lncRNA loci overlapping each set of selection regions was calculated using BEDTools (v2.31.1) intersect with the -u option, requiring at least 1 bp overlap. To generate a random genomic expectation, the genomic positions of lncRNA loci were randomly shuffled 10,000 times using BEDTools shuffle while preserving chromosome assignment and locus length distribution. The -chrom option was used to constrain each shuffled lncRNA locus to its original chromosome. For each permutation, the number of shuffled lncRNA loci overlapping the corresponding selection regions was recalculated using the same overlap criterion. Fold enrichment was calculated as the observed overlap divided by the mean overlap from the randomized datasets. Empirical *p*-values were calculated as (k + 1)/(*N* + 1), where k represents the number of permutations in which the randomized overlap was equal to or greater than the observed overlap, and N represents the total number of permutations.

### 4.4. Differential Expression Analysis

To identify the expression changes in lncRNAs and PCGs between the earlier (CN1960&70) and modern (CN2000&10) breeding eras, the raw count matrix was used as the input for DESeq2 v1.30.0, and fragments per kilobase of transcript per million mapped reads (FPKM) values were calculated to measure the expression levels of genes and transcripts in each IL [44]. DESeq2 internally normalizes raw counts using the median-of-ratios method and fits a negative binomial generalized linear model to the count data. Statistical significance was assessed using the Wald test, and the resulting *p*-values were adjusted for multiple testing using the Benjamini–Hochberg method to control the false discovery rate. Transcripts with |log_2_ (fold change)| ≥ 1 and adjusted *p*-value < 0.05 were defined as differentially expressed lncRNAs (DElncRNAs) or protein-coding genes (DEPCGs).

### 4.5. K-Means Clustering Analysis

To characterize the expression patterns of lncRNAs across three different breeding eras (CN1960&70s, CN1980&90s, and CN2000&10s), the standardized FPKM expression values of all expressed lncRNAs across three breeding periods were subjected to K-means clustering analysis using the kmeans function in R software (v4.0.2). The optimal number of clusters was determined using the Figure of Merit (FOM) module.

### 4.6. Prediction of Cis-Acting Target Genes for Differentially Expressed lncRNAs

Through cis-acting mechanisms, lncRNAs can influence the expression of adjacent genes. For each DElncRNA, potential cis-regulatory target genes were predicted based on the genomic distance to adjacent PCGs. Genomic coordinates of all DE-lncRNAs and PCGs were extracted from the maize genome annotation. Putative cis-target genes for each lncRNA were identified as those adjacent within 100 kb upstream or downstream using a Perl script [45]. Protein-coding genes located within 100 kbp upstream or downstream of each lncRNA were initially identified as candidate cis-regulatory targets. To reduce potential false positives introduced by distance-based prediction alone, Pearson correlation analysis was further performed between each candidate lncRNA–mRNA pair across all RNA-seq samples. Only pairs with |Pearson’s *r*| ≥ 0.6 and *p*-value < 0.05 were retained as high-confidence cis-regulatory pairs.

### 4.7. Weighted Gene Co-Expression Network Analysis (WGCNA)

By utilizing the WGCNA R framework (v1.70.3) [46], we constructed genetic networks from the 137 sample transcriptomic data to uncover modules linked with agronomic traits. An adjacency matrix was generated to determine the similarity among gene expressions, which was transformed through a soft thresholding procedure using the “pickSoftThreshold” function in the WGCNA R package. Using scale independence > 0.85 and connectivity < 100 as the criteria, a soft threshold power of 3 was selected as the lowest power that provided an acceptable approximation to a scale-free topology while retaining sufficient network connectivity (Figure S3). The adjacency matrix was then converted into a topological overlap matrix (TOM), and genes were hierarchically clustered based on TOM dissimilarity. Initial modules were identified using the dynamic tree cut algorithm with a minimum module size of 30. Modules with similar eigengenes were merged using a merge cut height of 0.25, corresponding to a module eigengene correlation of 0.75. Module eigengenes were subsequently correlated with eight phenotypic traits to identify candidate trait-associated co-expression modules. Because these analyses are based on expression correlations, the identified module–trait associations were interpreted as correlative rather than causal. Hub genes were defined as genes with high connectivity within the same module (absolute Module Membership ≥ 0.8) and significant correlation with the corresponding trait (absolute Gene Significance ≥ 0.2) [46].

To evaluate the robustness and stability of the co-expression modules identified by WGCNA, module preservation analysis was performed using the modulePreservation function in the WGCNA R package. The full expression dataset was randomly divided into a reference set and a test set, and the preservation of the original module structure was assessed between the two subsets. Preservation statistics were calculated with 200 permutations. Module preservation was evaluated using Zsummary and medianRank. Modules with Zsummary > 10 were considered strongly preserved, modules with 2 < Zsummary ≤ 10 were considered moderately preserved, and modules with Zsummary ≤ 2 were considered to show no evidence of preservation. Because medianRank is less dependent on module size, lower medianRank values indicate stronger relative preservation.

### 4.8. Construction of the Putative ceRNA-Associated Regulatory Network

The lncRNA-associated ceRNA network was constructed by integrating miRNA binding predictions with expression profile analysis. First, the sequences of known mature maize miRNAs were retrieved from the miRBase database (v22.1) [47]. Potential interactions between miRNAs and lncRNAs, as well as between miRNAs and mRNAs (PCGs), were predicted using the plant-specific tool psRNATarget [48], with a maximum expectation value of ≤3.0. Candidate lncRNA–miRNA–mRNA triplets were then identified by retaining lncRNAs and mRNAs that were both predicted to be targeted by the same mature maize miRNA. In addition, the expression levels of the lncRNA and mRNA were required to be significantly positively correlated (Pearson’s *r* > 0.4, *p*-value < 0.05). Finally, the resulting putative ceRNA-associated network was visualized using Cytoscape (v3.7.1). Because matched miRNA expression data were unavailable, this analysis was used to prioritize hypothetical candidate ceRNA-like associations rather than to infer validated ceRNA regulatory relationships.

### 4.9. Dual-Luciferase Reporter Assay in Nicotiana benthamiana

The interaction between lncRNAs (lncrna.33063 and lncrna.33068) and *ZmPIF5.2* was validated using a dual-luciferase reporter assay in *Nicotiana benthamiana* leaves. The full-length sequences of the candidate lncRNAs were cloned into the pBinGFP2 (Beyotime, Shanghai, China) vector under the control of the CaMV 35S promoter. For reporter constructs, approximately 1500 bp promoter fragments upstream of the translation start sites of *ZmPIF5.2* were amplified and inserted into the pGreenII 0800-LUC vector to drive firefly luciferase (LUC) expression [49]. The Renilla luciferase (REN) gene, driven by a constitutive 35S promoter within the same vector, served as an internal control. The recombinant constructs were transformed into *Agrobacterium tumefaciens* strain GV3101 (WeiDi, Shanghai, China). For transient expression, Agrobacterium cultures were resuspended in infiltration buffer (10 mM MES, 10 mM MgCl_2_, and 150 μM acetosyringone, pH 5.6) (Sigma-Aldrich, St. Louis, MO, USA) to an OD_600_ of 0.8. Equal volumes of different combinations were co-infiltrated into the abaxial surface of 4-week-old *N. benthamiana* leaves using a needleless syringe. After 48 h of incubation, luciferase signals were captured using a CCD imaging system (Sage Imaging System, China). Firefly luciferase (Luc) and Renilla luciferase (RLuc) activities were measured using the Dual-Luciferase Reporter Assay System (Promega, Madison, WI, USA) on a luminometer. The relative luciferase activity was calculated as the ratio of Luc to RLuc. Three independent biological replicates were performed for each treatment. Statistical significance was assessed by one-way ANOVA followed by Tukey’s honestly significant difference (HSD) test (*p* < 0.05).

### 4.10. Functional Enrichment Analysis

To systematically explore the biological roles corresponding to multiple gene subsets (cis-targets, WGCNA cluster genes, and subnetwork mRNAs). The AgriGO toolkit (https://systemsbiology.cau.edu.cn/agriGOv2/FAQ.php, accessed on 6 February 2026) was deployed to extract corresponding Gene Ontology (GO) terms [50], while KEGG pathway enrichment was resolved using the KOBAS web service (http://bioinfo.org/kobas, accessed on 10 February 2026) [51]. Significance was evaluated statistically using a hypergeometric testing framework, with terms and pathways defined as significantly enriched if their FDR-adjusted *p*-values were <0.05.

## Figures and Tables

**Table 1:** Overlap between identified lncRNA loci and selection signal regions.

**Selection Regions**	**lncRNAs Completely Contained**	**lncRNAs with Any Overlap (≥1 bp)**	**Mean Random Overlap**	**Fold Enrichment**	**Empirical *p*-Value**
CN1980&90 vs. CN1960&70	903	934	650.3240828	1.436207	0.009987
CN2000&10 vs. CN1960&70	1017	1076	776.8397782	1.385099	0.00987001
CN2000&10 vs. CN1980&90	799	846	566.4534989	1.493503	0.00029997
Ex-PVP vs. Public-US	1906	1935	2126.420361	0.90998	0.0959804

## Data Availability

The raw RNA-seq data have been deposited to the national center for biotechnology information (NCBI) SRA database under the Bioproject ID PRJNA783356.

## Supplementary Materials

The following supporting information can be downloaded at: https://www.mdpi.com/article/10.3390/plants15121772/s1, Figure S1: Systematic identification pipeline and stringent filtering of lncRNAs in maize; Figure S2: Structural features of identified maize lncRNA candidates; Figure S3: Determination of the soft-thresholding power for WGCNA network construction; Figure S4: Expression profiles of key WGCNA modules significantly associated with specific agronomic traits; Table S1: Coordinate-based overlap analysis between identified maize lncRNAs and PLncDB-annotated lncRNAs; Table S2: Differentially expressed lncRNAs identified between earlier and modern maize breeding eras; Table S3: Differentially expressed PCGs identified between earlier and modern maize breeding eras; Table S4: List of lncRNAs cis-target gene; Table S5: Pearson correlation analysis of lncRNA-mRNA pairs; Table S6: Module preservation statistics of the 33 WGCNA modules; Table S7: The relationship of miRNA with lncRNA and mRNA.
