# Supplementary material for: Genome-Wide Characterization of Long Non-Coding RNAs Identifies Candidate Regulatory Networks During Modern Maize Breeding
Source: Plants (Basel). 2026 Jun 8;15(12):1772. doi: 10.3390/plants15121772 (PMC13306248; doi:10.3390/plants15121772)
Supplement: Supplementary file 1 [file plants-15-01772-s001.zip › Supplementary figure.pdf]

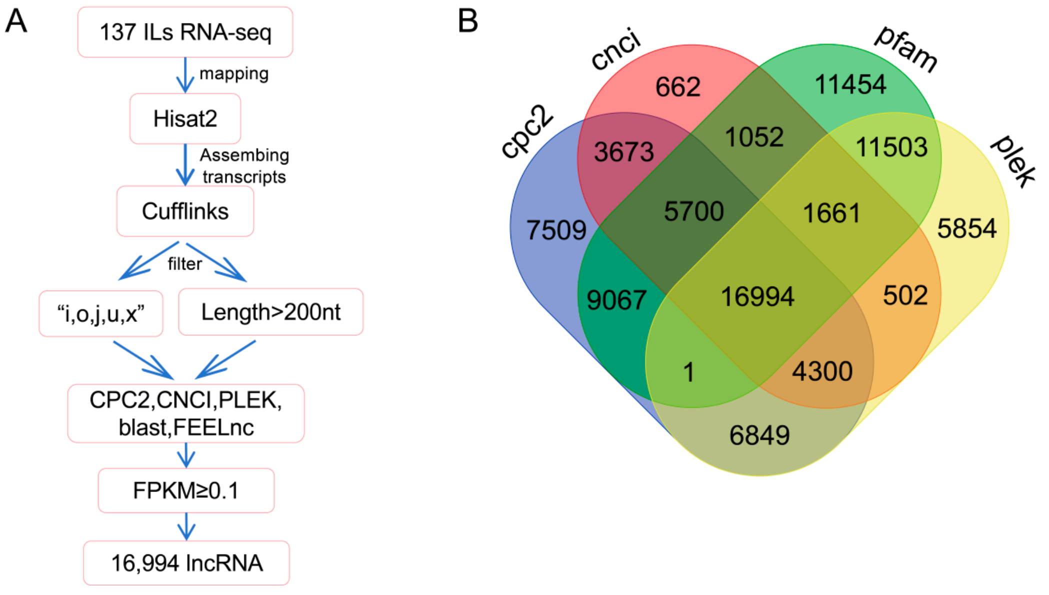

Figure S1. Systematic identification pipeline and stringent filtering of lncRNAs in maize.

(A) Bioinformatics pipeline for the genome-wide identification of lncRNAs. (B) Venn diagram showing the overlap of non-coding transcripts identified by four coding potential prediction tools.

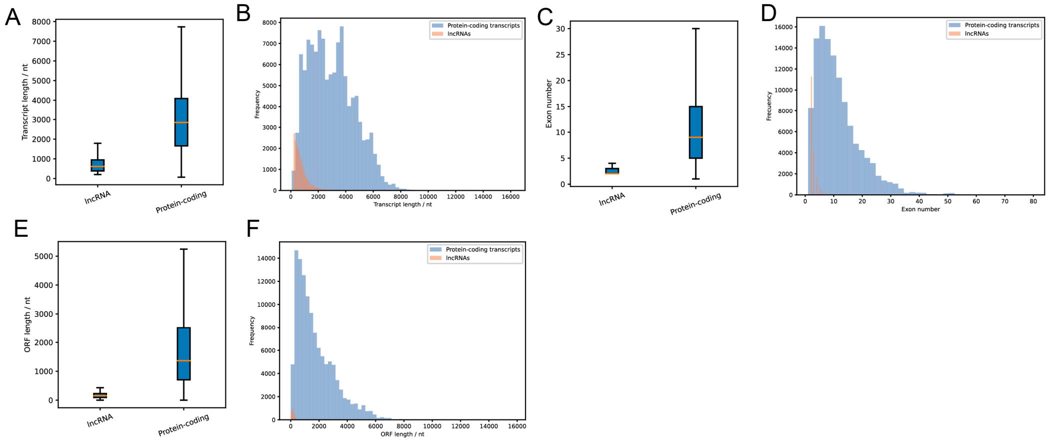

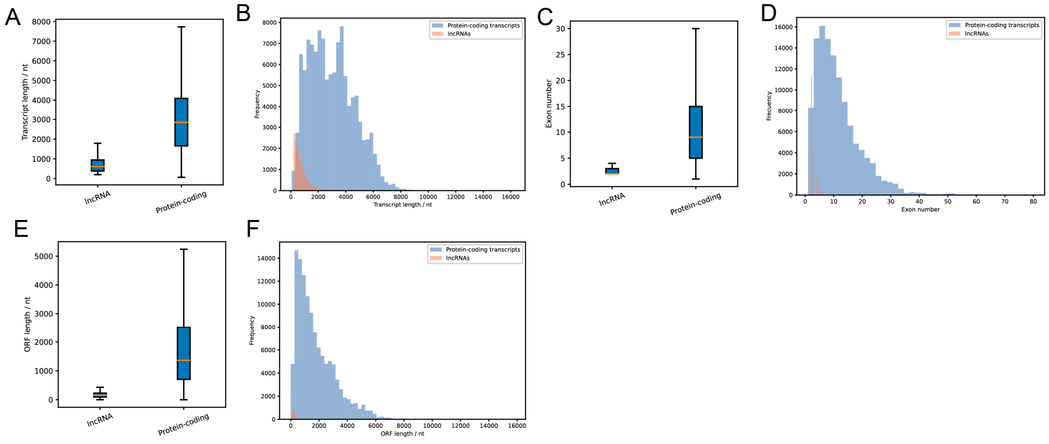

Figure S2. Structural features of identified maize lncRNA candidates.

(A) Boxplot of transcript length distribution. (B) Frequency distribution of transcript lengths. (C) Boxplot of exon number distribution. (D) Frequency distribution of exon numbers. (E) Boxplot of predicted ORF length distribution. (F) Frequency distribution of predicted ORF lengths. Protein-coding transcripts are shown in blue, and lncRNAs are shown in orange.

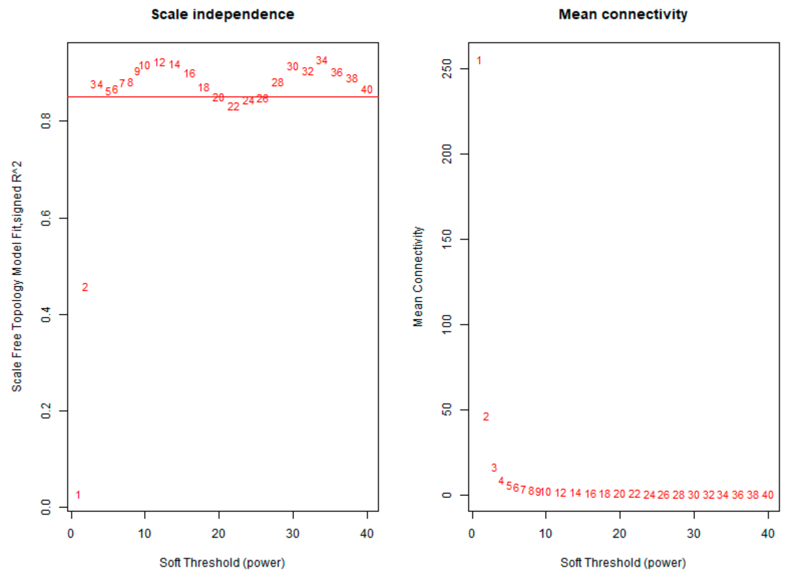

Figure S3. Determination of the soft-thresholding power for WGCNA network construction.

In the left panel, the scale-free topology model fit, signed R^2, is plotted against the soft-thresholding power. The right panel shows the corresponding mean connectivity as a function of soft-thresholding power.

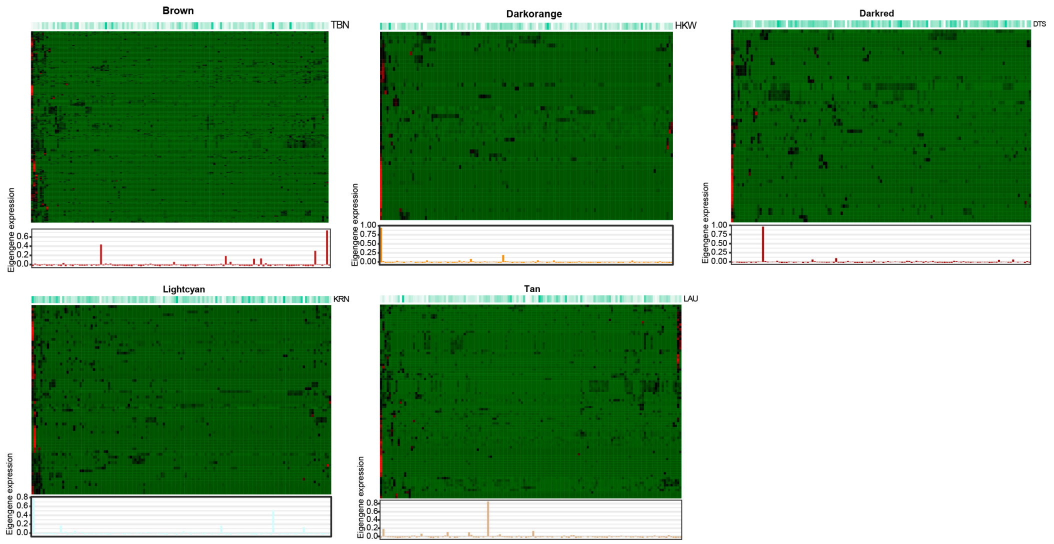

Figure S4. Expression profiles of key WGCNA modules significantly associated with specific agronomic traits.

Heatmaps and module eigengene expression bar plots are shown for five co-expression modules: Brown, Darkorange, Darkred, Lightcyan, and Tan. In each panel, the top heatmap displays the relative expression levels of all genes within the module across samples; the bottom bar plot illustrates the module eigengene values, representing the overall coordinated expression pattern of the genes within that module.
