# Supplementary figures and images for: Genome-Wide Characterization of Long Non-Coding RNAs Identifies Candidate Regulatory Networks During Modern Maize Breeding
Source: Plants (Basel). 2026 Jun 8;15(12):1772. doi: 10.3390/plants15121772 (PMC13306248; doi:10.3390/plants15121772)

### Scale independence

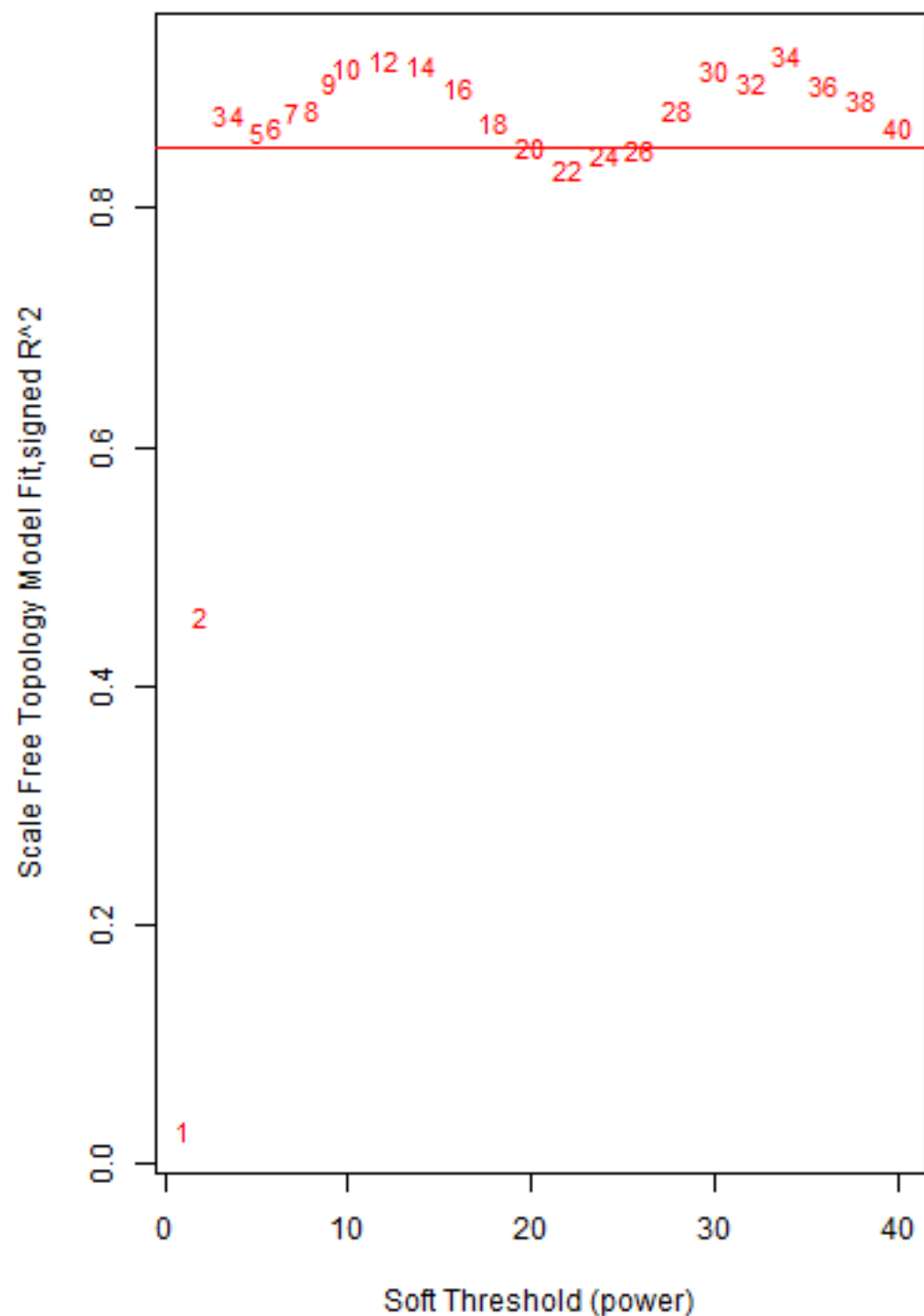

### Mean connectivity

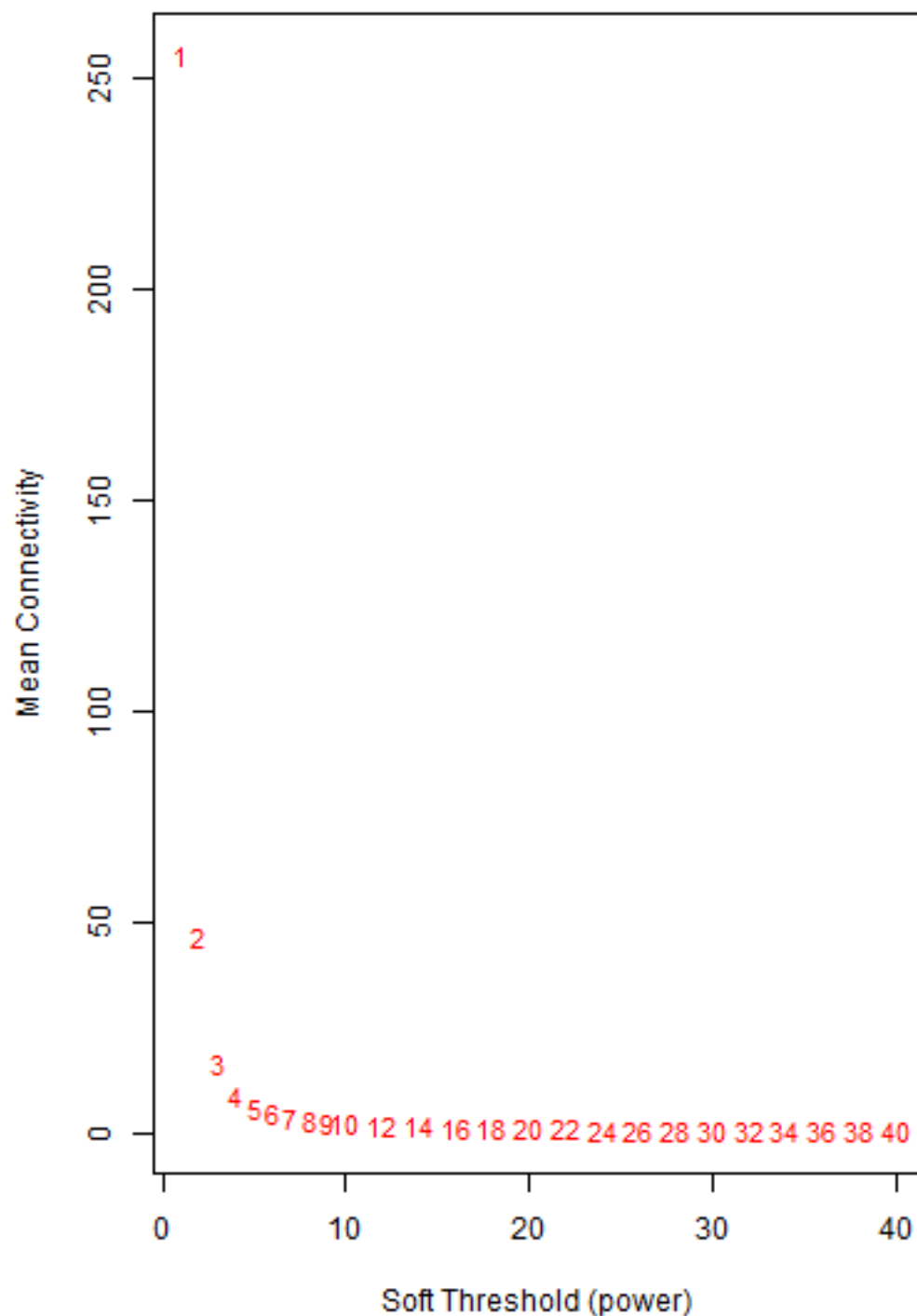

Supplement: Supplementary file 1 [file plants-15-01772-s001.zip › figureS3.pdf]

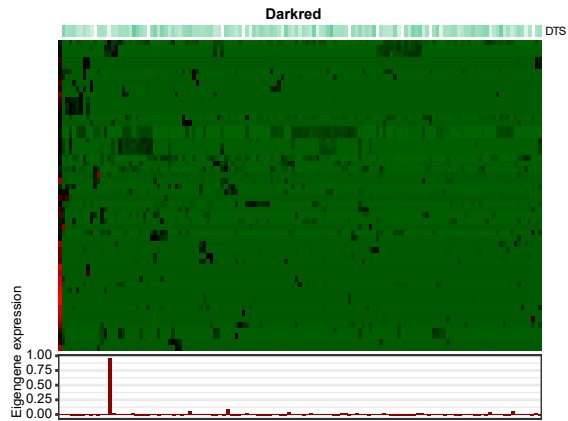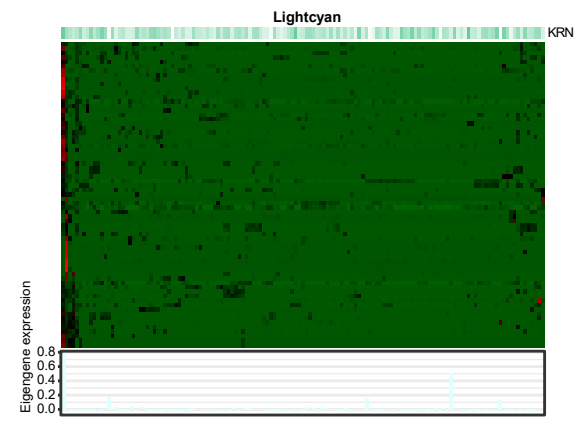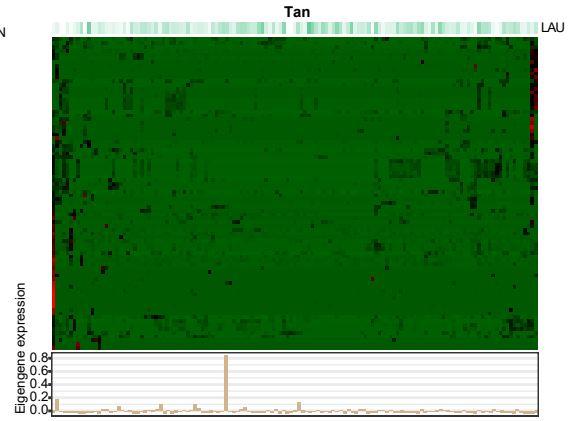

Supplement: Supplementary file 1 [file plants-15-01772-s001.zip › figureS4.pdf]

A

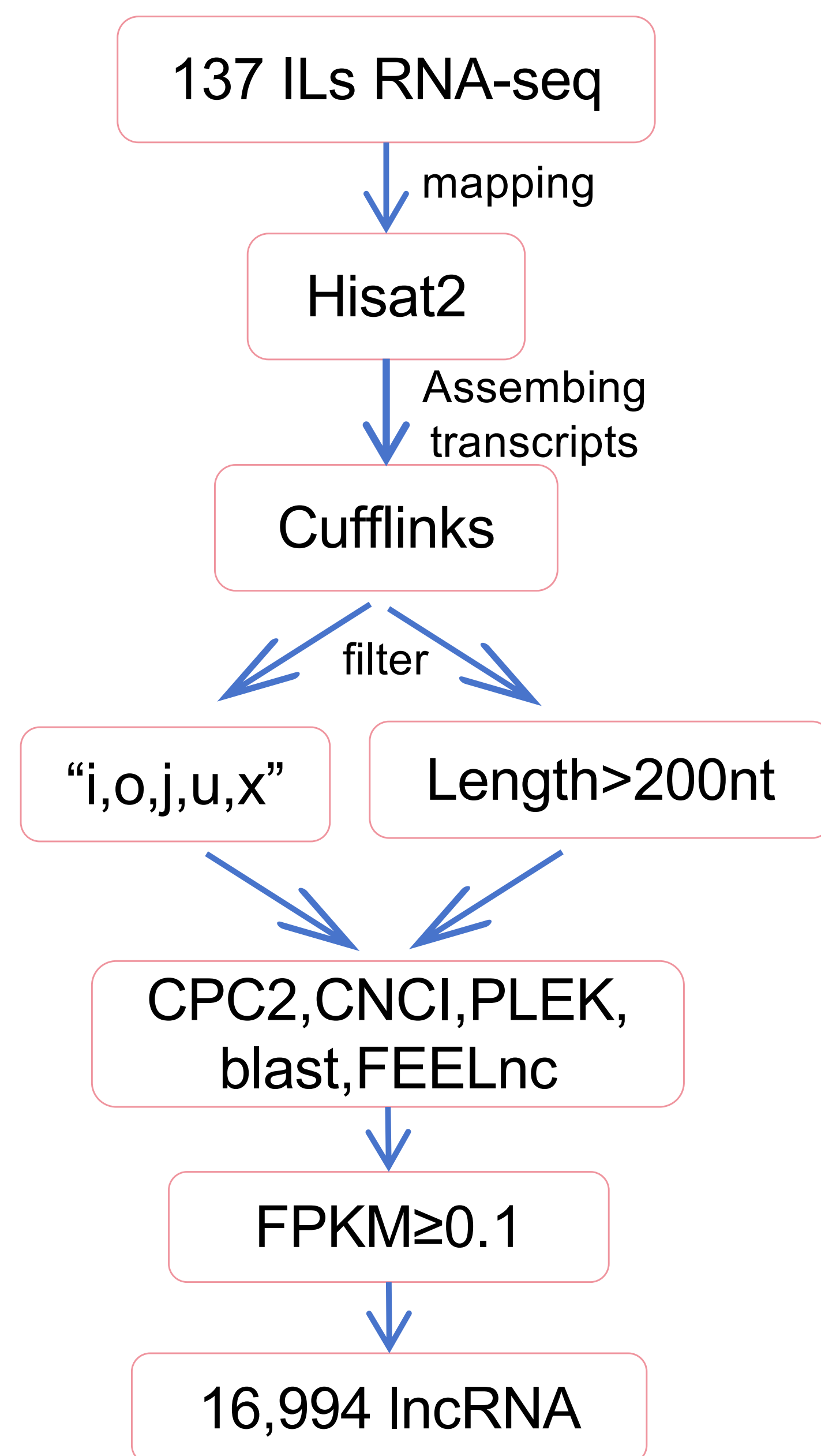

B

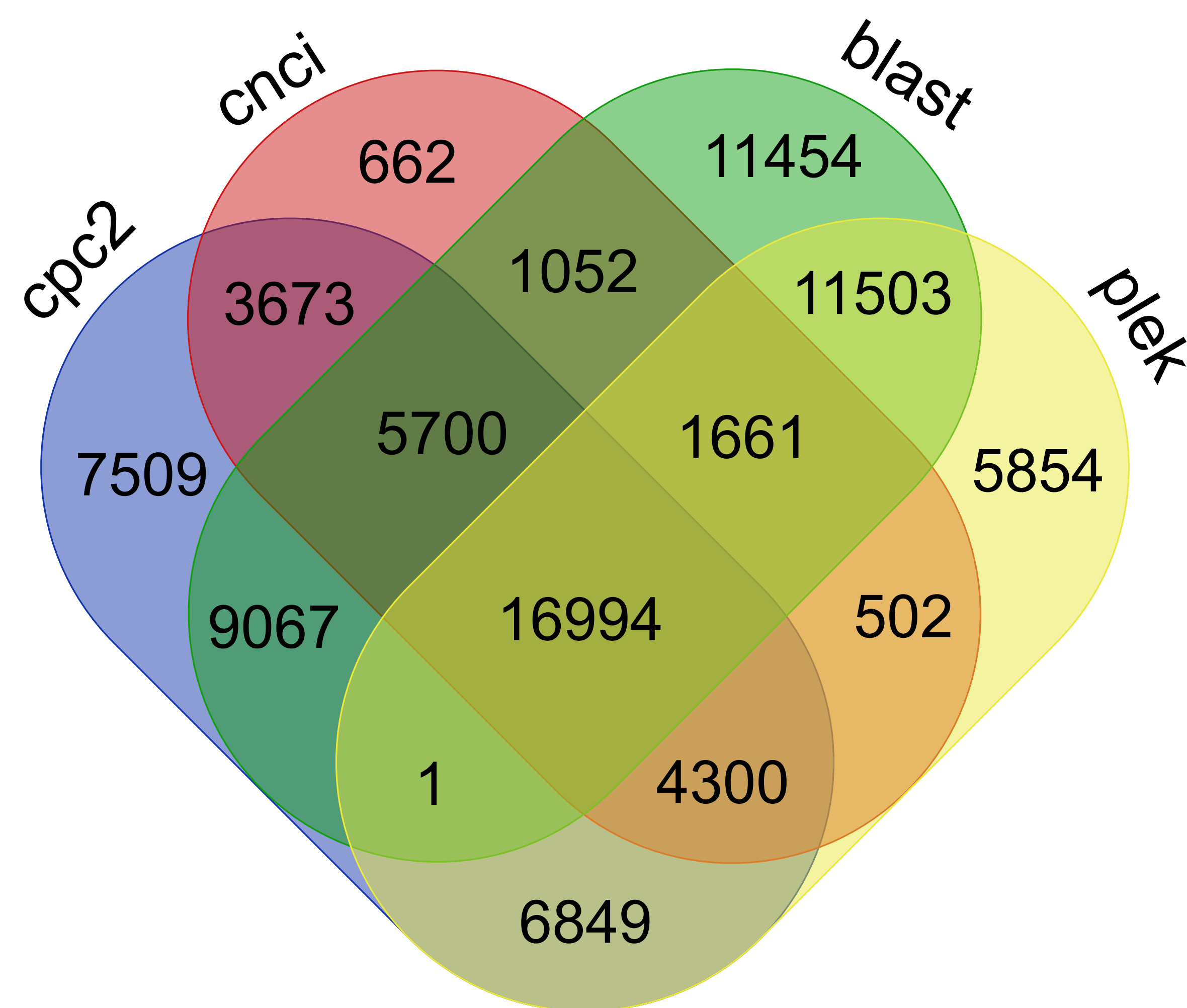

Supplement: Supplementary file 1 [file plants-15-01772-s001.zip › figureS1.pdf]

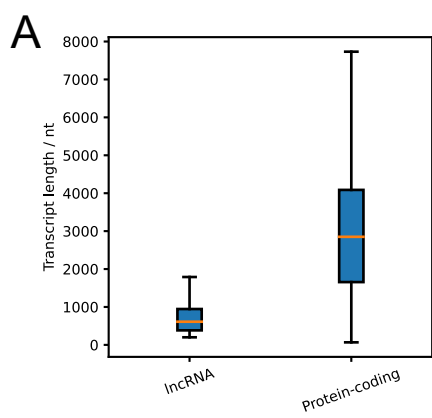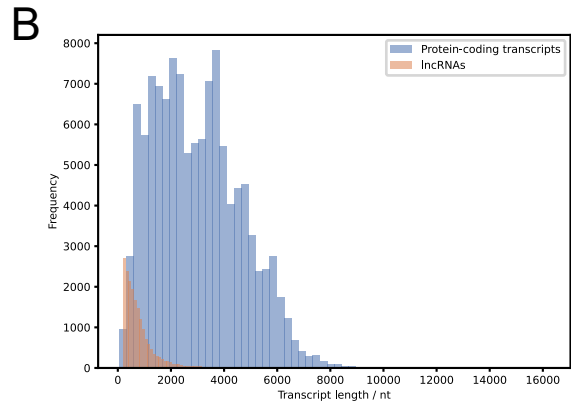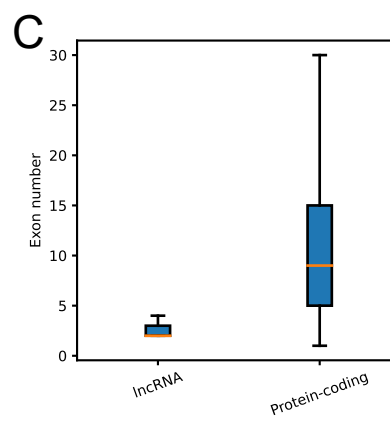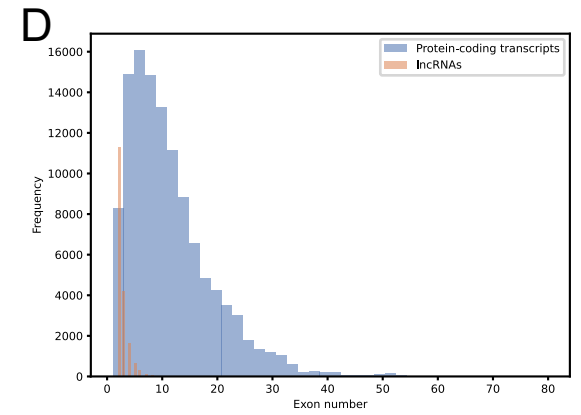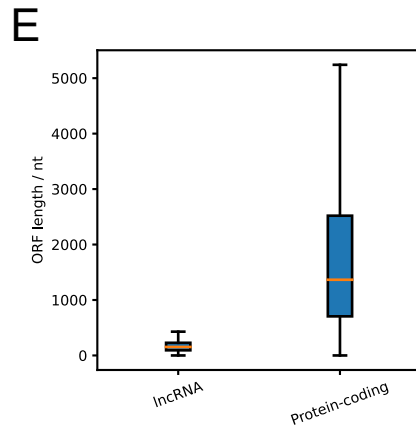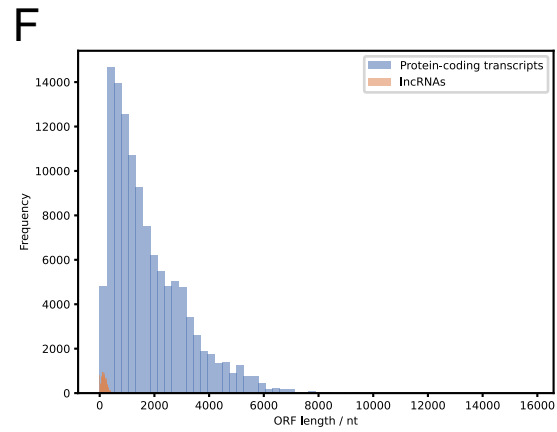

Supplement: Supplementary file 1 [file plants-15-01772-s001.zip › figureS2.pdf]
